# In vitro study of the trans-epithelial crossing of HIV-1 through the female genital mucosa and of the role of epithelial cells in the selection of CCR5-tropic virus

**DOI:** 10.1186/1742-4690-9-S1-P1

**Published:** 2012-05-25

**Authors:** R Terrasse, O Delezay, A Brunon-Gagneux, L Heyndrickx, H Hamzeh-Cognasse, B Pozzetto, T Bourlet

**Affiliations:** 1 Gimap Ea3064, Saint-Étienne, France

## Object

HIV heterosexual transmission mainly occurs by exposition of female genital mucosa with infected male seminal secretions. The notion of compartmentalization of HIV in the male genital tract is well established by the demonstration of the existence of several viral populations between blood and semen, especially in the gp160 viral envelope. The objectives of the present work were (i) the study of the role of genital epithelial cells in the heterosexual transmission of HIV and (ii) the in vitro modeling of this crossing by using chimeric viruses (pBrNL4.3-eGFP/dsRedExpress) that express the gp160 glycoprotein isolates from patients’semen.

## Methods

Seminal samples that showed a positive viral load were selected from 50 semen specimens obtained from 39 HIV-infected patients followed at the University-Hospital ofSaint-Etienne. Chimeric viruses were constructed by cloning a pBrNL4.3 vector with a viral envelope containing a GFP or a dsRedExpress fluorochrome. HEC genital epithelial cells were infected by CXCR4-tropic (LAI) or CCR5-tropic (BaL) HIV-1 viruses (0.001 MOI per cell during 24h) in different conditions (presence or absence of AZT or proinflammatory cytokines).

## Results

Two days after infection, intracellular viral RNA was detected for both virus strains (LAI and BaL) either in the presence or in the absence of AZT. By contrast, the detection of proviral DNA (LTR) was observed only for the CXCR4-tropic variant (500 ± 100 copies / 106 cells); the addition of AZT abolished this infection. Neither intracellular viral proteins nor extracellular viral RNA could be detected in infected cells.

## Conclusion

These results confirm the selective sequestration of CXCR4-tropic viruses by epithelial cells. Complementary analyses are ongoing for determining the exact role of these cells in HIV-1 heterosexual transmission and as a viral latency reservoir that could be reactivated under inflammatory conditions. A confocal microscopy imaging using the chimeric viruses will complete these preliminary results.

